# Updating the diagnostic criteria of COVID-19 “suspected case” and “confirmed case” is necessary

**DOI:** 10.1186/s40779-020-00245-9

**Published:** 2020-04-04

**Authors:** Yun-Yun Wang, Ying-Hui Jin, Xue-Qun Ren, Yi-Rong Li, Xiao-Chun Zhang, Xian-Tao Zeng, Xing-Huan Wang, for the Zhongnan Hospital of Wuhan University Novel Coronavirus Management and Research Team

**Affiliations:** 1 Center for Evidence-Based and Translational Medicine, Zhongnan Hospital of Wuhan University, Wuhan, 430071 Hubei China; 2 Institute of Evidence-Based Medicine and Knowledge Translation, Henan University, Kaifeng, 475000 Henan China; 3 Department of Clinical Laboratory, Zhongnan Hospital of Wuhan University, Wuhan, 430071 Hubei China; 4 Leishenshan Hospital of Wuhan, Wuhan, 430200 Hubei China; 5 Department of Radiology, Zhongnan Hospital of Wuhan University, Wuhan, 430071 Hubei China

**Keywords:** COVID-19, SARS-CoV-2, 2019-nCoV, Guideline, Prevention, Diagnosis, Treatment, Novel coronavirus

## Abstract

On 6 February 2020, our team had published a rapid advice guideline for diagnosis and treatment of 2019 novel coronavirus (2019-nCoV) infection, and this guideline provided our experience and make well reference for fighting against this pandemic worldwide. However, the coronavirus disease 2019 (COVID-19) is a new disease, our awareness and knowledge are gradually increasing based on the ongoing research findings and clinical practice experience; hence, the strategies of diagnosis and treatment are also continually updated. In this letter, we answered one comment on our guideline and provided the newest diagnostic criteria of “suspected case” and “confirmed case” according to the latest Diagnosis and Treatment Guidelines for COVID-19 (seventh version) that issued by the National Health Committee of the People’s Republic of China.

Dear Editor,

In December 2019, the 2019 novel coronavirus (2019-nCoV) has caused an outbreak, which is now officially named as the coronavirus disease 2019 (COVID-19) and the virus has been named severe acute respiratory syndrome coronavirus 2 (SARS-CoV-2). On 11 March 2020, WHO characterized COVID-19 as a pandemic [1]. In order to fight against the SARS-CoV-2 infection, our team has developed a rapid advice guideline and that has been published online in *Military Medical Research* on 06 February 2020 [2]. It has attracted a great attention since published. Note however that COVID-19 is a new disease, our awareness and knowledge is gradually increasing based on the ongoing research findings and clinical practice experience [3–8]; hence, the strategies of diagnosis and treatment are also continually updated. For instance the *Diagnosis and Treatment Guidelines for COVID-19* issued by the National Health Committee of the People’s Republic of China (http://www.nhc.gov.cn/), among 16 January 2020 to 3 March 2020, has issued a total of seven editions with some contexts being substantively changed.

Now our guideline received a comment by Zhou et al. [9], they introduced a simple scoring proposal based on their clinical experience. Their work added new evidence for our guideline and also make valuable reference for this pandemic worldwide. We endorse their significant work and express our thanks. However, their work also needs update according to the latest *Diagnosis and Treatment Guidelines for COVID-19* (Trial seventh version) [10] and recently studies.

According to the seventh edition (3 March 2020), to confirm the suspected case needs to combine any one item of epidemiological history features with two items of clinical manifestations to make a comprehensive analysis, or needs to meet three items of clinical manifestations if without clear epidemiological history:

- Epidemiological history: (1) a history of travel or residence in Wuhan city and surrounding areas, or other communities where COVID-19 cases had been reported in the last 14 days before symptom onset; (2) a history of contact with SARS-CoV-2 infectious cases (with positive nucleic acid test); (3) a history of contacting with patients with fever or respiratory symptoms from Wuhan city and surrounding areas, or other communities where COVID-19 had been reported in the last 14 days before symptom onset; (4) a history of contacting with cluster of confirmed cases (≥ 2 cases with fever and/or respiratory symptoms occurred within 2 weeks in small areas, such as home, office, class of school, etc).
- Clinical manifestations: (1) fever and/ or respiratory symptoms; (2) with imaging features of COVID-19 infection; (3) total white blood cell counts showing normal, decreased, or reduced lymphocyte count in the early onset stage.

Diagnosing the confirmed case should base on suspected case with any one item of pathogenic or serological evidence as following: (1) real-time PCR test positive for SARS-CoV-2; (2) viral whole genome sequencing showing high homogeneity to the known novel coronaviruses; (3) positive for the specific IgM antibody and IgG antibody to SARS-CoV-2 in serum test; or a change of the SARS-CoV-2-specific IgG antibody from negative to positive, or titer rising ≥4 times in the recovery phase above that in the acute phase.

We can see that the real-time PCR test for nucleic acid in respiratory tract or blood samples was added to the second (18 January 2020) and third (22 January 2020) editions. The pathogenic detection of blood sample was added to the fourth (27 January 2020) and fifth (8 February 2020) editions; and then the serological evidence was added to the seventh edition. These modifications based on the researchers continued work that to search for an optimal nucleic acid detection kit for rapid diagnosis, as well as the samples from respiratory tract including blood sampling, which increased the availability of different specimens, and supported bringing the specific antibody positive result into the confirmed criteria [11–14].

Besides, there are more and more evidence that remind us to caution with the atypical symptomatic and asymptomatic patients [13, 15–18]. Hence, the flow chart of Zhou et al. [9] should be updated, as they classified the person without clinical symptoms as “low risk”. The score system also needs to be verified in further clinical practice and studies.

To conclude, we hope more direct evidence coming up and call for readers to provide their comments. For the diagnosis of “suspected case” and “confirmed case”, we suggest to trace and obey the newest guidelines of their home countries. Our team will also timely update our guideline to offer help.

## Data Availability

Not applicable.

## Abbreviations

2019-nCoV: 2019 novel coronavirus
COVID-19: Coronavirus disease 2019
SARS-CoV-2: Severe acute respiratory syndrome coronavirus 2
